# Association between physical performance during sit-to-stand motion and frailty in older adults with cardiometabolic diseases: a cross-sectional, longitudinal study

**DOI:** 10.1186/s12877-023-04011-z

**Published:** 2023-05-30

**Authors:** Yuji Murao, Joji Ishikawa, Yoshiaki Tamura, Fumino Kobayashi, Ai Iizuka, Ayumi Toba, Kazumasa Harada, Atsushi Araki

**Affiliations:** 1 Center for Comprehensive Care and Research for Prefrailty, Tokyo Metropolitan Institute for Geriatrics and Gerontology, 35-2 Sakaecho, Itabashi-Ku, Tokyo, 173-0015 Japan; 2 Department of Cardiology, Tokyo Metropolitan Institute for Geriatrics and Gerontology, Tokyo, Japan; 3 Department of Diabetes, Metabolism, and Endocrinology, Tokyo Metropolitan Institute for Geriatrics and Gerontology, Tokyo, Japan

**Keywords:** Frailty, Physical performance, Ground reaction force, Cardiometabolic disease

## Abstract

**Background:**

Although physical performance tests of the lower extremities are used to assess sarcopenia and frailty, little is known about the mechanisms by which the parameters of ground reaction force (GRF) measured during sit-to-stand motion affect the frailty status in older adults. We aimed to examine the association between GRF parameters during sit-to-stand motion and the incidence of frailty in older adults.

**Methods:**

This longitudinal study evaluated 319 outpatients aged ≥ 65 years with cardiometabolic diseases. The GRF parameters were measured using a motor function analyzer, in which the power, speed, and balance scores were calculated. Frailty was diagnosed using the modified version of the Cardiovascular Health Study (mCHS) and the Kihon Checklist (KCL). The independent associations between scores and frailty indices were assessed using multivariate binomial logistic regression analyses. Cox regression analysis was used to examine whether power and speed scores were associated with the incidence of frailty after adjusting for covariates.

**Results:**

Logistic regression analyses adjusted for covariates showed that the power and speed scores were associated with frailty according to the mCHS criteria (power: OR = 0.37, 95% CI = 0.22–0.63; speed: OR = 0.64, 95% CI = 0.52–0.79) and KCL criteria (power: OR = 0.40, 95% CI = 0.26–0.62; speed: OR = 0.81, 95% CI = 0.69–0.96) at baseline. Receiver operating characteristic analyses revealed that the area under the curve values of power and speed scores for discriminating mCHS-defined frailty were 0.72 and 0.73. The Cox regression analysis showed that the speed score predicted the incidence of mCHS-defined (HR = 0.45, 95% CI = 0.22–0.92, *P* = 0.029) and KCL-defined (HR = 0.77, 95% CI = 0.60–0.99, *P* = 0.039) frailty, whereas the power score was associated with the incidence of KCL-defined frailty (HR = 0.72, 95% CI = 0.55–0.95, *P* = 0.02) after adjusting for covariates.

**Conclusions:**

The speed and power scores measured during sit-to-stand motion are predictive of frailty in older adults with cardiometabolic disease. Therefore, the GRF parameters measured during sit-to-stand motion could be an important indicator of frailty. Further studies are necessary to examine whether the GRF parameters can be improved by exercise or whether the changes in these parameters are associated with the improvement of frailty status.

**Supplementary Information:**

The online version contains supplementary material available at 10.1186/s12877-023-04011-z.

## Background

Population aging is expected to progress rapidly, not only in developed regions but also on a global scale, including the developing regions [1]. Thus, extending the well-being and healthy life expectancy of older adults has become an important issue. Frailty has become an important concept related to extending healthy life expectancies as it is characterized by decreased physiologic reserve, increased vulnerability to stressors, and a high risk of adverse health outcomes such as disability or mortality [2]. Therefore, early detection during the reversal stage and taking preventive measures against frailty are important for older adults.

Diabetes and cardiovascular diseases are associated with frailty [3, 4]. Since patients who concurrently develop cardiometabolic diseases and frailty are highly susceptible to functional disability and death, it is highly important to identify who are frail or at high risk of frailty in this population. However, no reference standards have been established for measuring frailty in older patients with cardiovascular metabolic risk factors in the clinical setting. The Cardiovascular Health Study (CHS) criteria [5], Kihon Checklist (KCL) [6], Clinical Frailty Scale [7], and Frail Scale [8] have been used to assess frailty. Various versions of the CHS criteria have been developed, and the Japanese version [9, 10] and modified versions (mCHS) [11] of the physical frailty assessment criteria have been published. The Japanese version of the Cardiovascular Health Study (J-CHS) criteria have been proposed by Satake et al. [9, 10] and consist of the following items: 1) weight loss, 2) muscle weakness, 3) fatigue, 4) walking speed, and 5) physical activity. The mCHS criteria also consist of almost the same items as those in the J-CHS criteria [11]. The KCL is a well-validated frailty index based on the Comprehensive Geriatric Assessment (CGA) tool, which was originally developed as a screening method to identify older people at high risk of requiring nursing care.

Some physical performance tests, such as walking speed and the Timed Up and Go (TUG) test, are also utilized for frailty assessment owing to their good predictive ability [12–14]. However, the accurate measurement of walking speed and TUG time requires appropriate location and adequate space, which may be difficult to perform in some facilities. Physical performance tests are usually assessed by performing standing movements, such as the five-consecutive chair rise test. However, despite being an easy procedure, it is difficult to determine which dynamic parameters of the sit-to-stand motion test are related to frailty.

The ground reaction force (GRF) during the sit-to-stand motion is measured using a motor function analyzer [15]. The maximum GRF/body weight (F/wt), rate of force development/body weight (RFD/wt), and lateral load sway during sit-to-stand motion are used to indicate power, speed, and balance of physical performance, respectively. The power score (F/wt) is associated with disabilities in performing the activities of daily living [16] and the incidence of falls [15], while the speed score (RFD/wt) is related to the TUG time and walking speed [14]. Low power, speed, and balance scores are associated with high serum growth differentiation factor-15 levels [17]; however, a few studies have reported the association between the dynamic parameters in sit-to-stand motion and incidence of frailty. In this study, we aimed to investigate the association between various dynamic parameters detected by motor function analyzer and the prevalence and incidence of frailty defined by two different concepts (physical and multimodal) in a cohort of old outpatients with cardiometabolic diseases.

## Methods

### Study design and participants

**Fig. 1:**
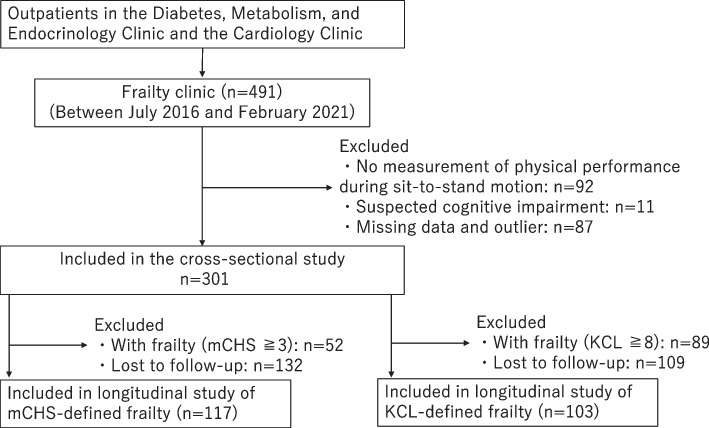
Flowchart of the patient selection process

This cross-sectional, longitudinal study aimed to investigate the association between the dynamic parameters of sit-to-stand motion and the two types of frailty in older adults with cardiometabolic diseases (hypertension, diabetes mellitus, dyslipidemia, atrial fibrillation, ischemic heart disease, and heart failure). The protocol of this study was reported in a previous study [11]. In total, 491 outpatients aged ≥ 65 years with cardiometabolic diseases and who visited our frailty clinic between July 2016 and February 2021 were evaluated. Of them, 399 participants underwent physical performance measurements during the sit-to-stand motion test, while 11 participants with suspected dementia were excluded from the study. Suspected dementia was defined as a Mini-Mental State Examination (MMSE) [18, 19] scale score of ≤ 23 or a Revised Hasegawa Dementia Scale (HDS-R) [20] score of ≤ 20. After further excluding patients with missing data, only 319 participants were included in the final analysis. With the time of enrollment used as the baseline, a longitudinal study was conducted in participants who had not been frail at baseline, based on the criteria described below. In the longitudinal study, 117 and 103 participants with mCHS-defined and KCL-defined frailty, respectively, were included in the analysis. Figure 1 shows the participant inclusion/exclusion process.

### Assessment of frailty, muscle mass, and physical performance

The mCHS criteria were used as an index of physical frailty, and five factors were assessed: weight loss, fatigue, low physical activity, low walking speed, and muscle weakness. Weight loss was confirmed if the participant responded “Yes” to the question, “Did you experience > 2–3 kg of weight loss in the last 6 months?” Fatigue was assessed using the question “In the last 2 weeks, have you felt tired without a reason?” Low physical activity was confirmed if the participant responded “No” to the question “Do you go out at least once a week?” or “Yes” to the question “Do you go out less frequently than you did last year?” These four questions were also included in the KCL question set. Low walking speed was defined as < 1.0 m/s in the 4-m walk tests [21]. The participants were asked to walk 6 m at normal speed, and the time spent to walk 4 m was measured using a stopwatch. The measurements were taken twice, and the larger value was used for the analyses. Muscle weakness was defined as handgrip strengths of < 28 kg for men and < 18 kg for women [21]. Grip strength was measured once on each side using a digital grip strength meter (T.K.K. 5401 GRIP-D; Takei Kiki Kogyo Co., Ltd.), and the larger value obtained after measuring both sides was used for the analyses. Frailty was defined as an mCHS index score of ≥ 3, while absence of frailty was defined as a score of ≤ 2. For the KCL, the ability to perform the instrumental activities of daily living, nutritional status, oral function, social function, cognition, and depression status were measured. Frailty was defined as a KCL score of ≥ 8 [22].

SMI was measured by performing a bioelectrical impedance analysis using a body composition analyzer (MC-780A-N; Tanita Corporation, Japan). The TUG test was used to identify the maximum walking speed by measuring the time taken by the participant to get up from a chair, walk 3 m away until the marker is reached, turn around, walk back to the chair, and sit down.

### Measurement of physical performance in sit-to-stand motion

To measure the physical performance during sit-to-stand motion, the participant stood up from a chair and performed three trials with approximately 2 s of rest in the standing position and 2 s of rest intervals in the sitting position. The participants were asked to cross their arms in front of their chests and stand up as quickly as possible. The trials in which the participants obtained the highest power, speed, and balance scores were recorded. The power score was defined as the maximum GRF divided by wt. The speed score was defined as the RFD at 87.5 ms divided by wt.

Balance score was defined as the T-score of the lateral load sway divided by the vertical load change per second when the highest RFD8.75/wt was recorded. Lateral load sway was calculated as the time from the point when the reaction force reached its peak in a stable standing posture. The total score was calculated using the Z-scores of the speed, power, and balance. Body composition, along with the resistance to reactance ratio, was measured using a body composition meter (Tanita MC-780A), with 75 points as the average score. The intraclass correlation coefficients for power, speed, and balance scores were considered good when they exceeded 0.7 [23, 24].

### Assessment of other variables

Data of patients’ medical history and comorbidities (dyslipidemia, hypertension, diabetes mellitus, heart failure, and stroke) were collected from the medical records, while data of the patients’ lifestyle behaviors (drinking habits and smoking status) were obtained by interview. Cognitive assessment was performed using the MMSE and HDS-R.

### Statistical analysis

The participants were divided into the frail and non-frail groups based on the mCHS and KCL scores. Age, body mass index (BMI), total score, power, speed, balance, walking speed, grip strength, and SMI were confirmed to be equally distributed using Levane’s test. The normally distributed variables were subjected to an uncorrelated t-test, while the non-normally distributed variables were subjected to Welch’s test. The χ^2^ test was performed for between-group comparisons of the prevalence of sex, smoking status, alcohol consumption, and comorbidities. Next, multivariate binomial logistic regression analysis was conducted using the mCHS- or KCL-defined frailty criteria as the dependent variables and power, speed, balance, and total scores as the independent variables. Age, sex, BMI, dyslipidemia, diabetes, hypertension, heart failure, stroke, smoking status, and alcohol consumption were used as covariates.

To examine the ability of power, speed, balance, and total score to distinguish the presence of frailty according to the mCHS and KCL criteria, receiver operating characteristic (ROC) analyses were performed, and the cutoff values were identified by calculating the area under the curve (AUC) and Youden index. The Youden index was calculated using the sensitivity and specificity values obtained by a statistical software. In addition, the AUC values of SMI, grip strength, walking speed, and TUG were calculated and compared with those of the total score, power score, speed score, and balance score obtained during the physical performance test. For the TUG test, the observed values were considered negative.

In the longitudinal study, the patients were further divided into two groups based on the cutoff point calculated using the Youden index as a reference. The frailty-free survival curves of two groups with power and speed scores below and above the cutoff levels were depicted using the multivariate Cox regression analyses and compared using the log-rank test. Finally, Cox regression analyses were used to examine whether the dynamic parameters during standing (power, speed, balance, and total scores) predicted the incidence of frailty, with participants who were free of frailty at baseline included in this analysis. In the multivariate Cox regression analysis, age, sex, BMI, dyslipidemia, diabetes, hypertension, heart failure, stroke, smoking status, and alcohol consumption were used covariates. All statistical analyses were performed using IBM SPSS Statistics version 25 (Chicago, USA). A *P* value of < 0.05 was considered significant.

## Results

### Patients’ characteristics

Among the 301 patients, 52 (17.2%) and 89 (30.0%) patients were classified as frail according to the mCHS and KCL criteria, respectively (Table 1). Age was significantly higher in the frail group (*P* < 0.01). SMI was lower in patients with KCL-defined frailty (*P* = 0.016). No significant differences were observed in sex, smoking status, alcohol consumption, MMSE scores, and the prevalence of comorbid diseases between the two groups. In both the mCHS and KCL criteria, the frail group had significantly lower power, speed, and balance scores; grip strength; walking speed; and longer TUG time.

**Table 1:** Patients’ baseline characteristics

Variables	Total	mCHS criteria			KCL criteria		
	(*n* = 301)	Frail	Non-frail		Frail	Non-frail	
		(*n* = 52)	(*n* = 249)	*P*	(*n* = 89)	(*n* = 212)	*P*
Age, years	78.7 ± 5.9	80.7 ± 6.2	78.3 ± 5.8	0.008	80.8 ± 6.4	77.8 ± 5.5	< 0.001
Women, %	202 (67.1)	40 (76.9)	162 (65.1)	0.098	63(70.9)	139(65.6)	0.379
BMI, kg/m^2^	23.4 ± 3.6	23.3 ± 4.3	23.4 ± 3.5	0.851	22.8 ± 3.9	23.6 ± 3.5	0.091
Alcohol, %	69 (22.9)	11 (21.2)	58 (23.3)	0.739	21 (23.6)	48 (22.6)	0.857
Smoking, %	14 (4.7)	2 (3.8)	12 (4.8)	0.762	4 (4.5)	10 (4.7)	0.933
Dyslipidemia, %	176 (58.5)	30 (57.7)	146 (58.6)	0.900	53 (61.6)	123 (58.0)	0.806
Diabetes mellitus, %	146 (48.5)	26 (50.0)	120 (48.2)	0.813	42 (47.2)	104 (49.1)	0.768
Hypertension, %	223 (74.1)	34 (65.4)	189 (75.9)	0.115	67 (75.3)	156 (73.6)	0.759
Heart failure, %	21 (7.0)	4 (7.7)	17 (6.9)	0.824	8 (9.0)	13 (6.1)	0.375
Stroke, %	26 (8.6)	4 (8.0)	22 (8.8)	0.790	7 (7.9)	18 (8.5)	0.858
Total score, points	60.8 ± 12.5	55.0 ± 11.4	62.0 ± 12.4	< 0.001	56.3 ± 12.2	62.6 ± 12.2	< 0.001
Power	1.2 ± 0.1	1.2 ± 0.1	1.2 ± 0.1	< 0.001	1.2 ± 0.1	1.2 ± 0.1	< 0.001
Speed	7.5 ± 1.7	6.4 ± 1.6	7.7 ± 1.6	< 0.001	7.0 ± 1.7	7.7 ± 1.7	0.001
Balance	43.9 ± 11.3	39.6 ± 10.4	44.8 ± 11.3	0.003	41.1 ± 11.7	45 ± 10.9	0.005
Walking speed, m/s	1.15 ± 0.26	0.9 ± 0.2	1.2 ± 0.2	< 0.001	1.0 ± 0.2	1.2 ± 0.2	< 0.001
Grip strength, kg	22.3 ± 6.8	19.1 ± 6.5	23.0 ± 6.7	< 0.001	20.2 ± 6.5	23.2 ± 6.8	0.001
SMI, kg/m^2^	6.8 ± 1.2	6.6 ± 1.2	6.9 ± 1.1	0.386	6.5 ± 1.2	6.9 ± 1.1	0.016
TUG (s)	8.6 ± 4.1	10.4 ± 3.4	8.2 ± 4.1	< 0.001	9.9 ± 5.5	8.0 ± 3.2	< 0.001
MMSE, points	27.8 ± 2.1	28.0 ± 1.9	27.9 ± 2.2	0.698	27.6 ± 2.06	28.0 ± 2.2	0.208

Data are expressed as mean ± standard deviation. *P* values are calculated using Student’s t-test or chi-square test

*mCHS* modified version of Cardiovascular Health Study, *KCL* Kihon Checklist, *BMI* body mass index, *SMI* skeletal muscle index, *TUG* Timed Up and Go test, *MMSE* Mini Mental State Examination

### Multivariate binominal logistic regression analysis of the association of power, speed, and balance with frailty at baseline

In the logistic regression analysis using mCHS-defined frailty as the dependent variable, the power, speed, and balance scores were significantly associated with mCHS-defined frailty in the crude model and the model adjusted for covariates (Table 2). Every 0.1-point increase in the power and speed scores, the mCHS-defined frailty odds ratios decreased by 63% and 36%, respectively. The total score was significantly associated with the prevalence of mCHS-defined frailty. In the logistic regression analysis using KCL-defined frailty as the dependent variable, the power, speed, and total scores were significantly associated with frailty, independent of the covariates (Table 2).

**Table 2:** Multivariate logistic regression analysis of frailty according to the physical performance scores in the sit-to stand motion test at baseline

Variables	Crude model		Multivariate model	
	Odds ratio (95% CI)	*P*	Odds ratio (95% CI)	*P*
**Model for frailty defined based on the mCHS criteria**				
Power score, per 0.1-point increase	0.35 (0.22–0.56)	< 0.001	0.37 (0.22–0.63)	< 0.001
Speed score, per 1- point increase	0.61 (0.50–0.75)	< 0.001	0.64 (0.52–0.79)	< 0.001
Balance score, per 1- point increase	0.96 (0.94–0.99)	0.004	0.97 (0.94–0.99)	0.014
Total score, per 10-points increase	0.64 (0.50–0.82)	< 0.001	0.65 (0.49–0.86)	0.003
**Model for frailty defined based on the KCL criteria**				
Power score, per 0.1-point increase	0.43 (0.30–0.63)	< 0.001	0.40 (0.26–0.62)	< 0.001
Speed score, per 1-point increase	0.77 (0.67–0.90)	0.001	0.81 (0.69–0.96)	0.016
Balance score, per 1-point increase	0.97 (0.95–0.99)	0.006	0.98 (0.96–1.00)	0.071
Total score, per 10-points increase	0.66 (0.54–0.82)	< 0.001	0.72 (0.56–0.91)	0.006

Multivariate model is adjusted for age, sex, BMI, dyslipidemia, diabetes, hypertension, heart failure, stroke, habitual drinker, and smoking

*CI* confidence interval, *KCL* Kihon Checklist, *mCHS* modified version of the Cardiovascular Health Study, *BMI* body mass index

### ROC analysis of the cut-off levels of power and speed scores for the association with frailty at baseline

The AUC values of the power and speed scores for discriminating mCHS-defined frailty were 0.72 and 0.73, respectively (Table 3 and Supplementary Fig. 1a), and these values were similar to those of grip strength and TUG time, slightly lower than those of walking speed, and higher than those of SMI. In the ROC analysis for discriminating KCL-defined frailty, the AUC value of the power score was 0.68, thus indicating a satisfactory discriminative power. This value was similar to that of walking speed and TUG (Table 3, Supplementary Fig. 1b).

**Table 3:** AUC of each variable for discrimination of frailty defined based on the mCHS and KCL criteria at baseline

	mCHS-defined frailty					KCL-defined frailty				
Variables	AUC	95% CI	Cut-off point	Sensitivity	Specificity	AUC	95% CI	Cut-off point	Sensitivity	Specificity
Power score	0.72	(0.63–0.80)	1.16	0.76	0.64	0.68	(0.61–0.75)	1.16	0.77	0.52
Speed score	0.73	(0.56–0.81)	6.92	0.73	0.69	0.64	(0.57–0.70)	7.13	0.67	0.60
Balance score	0.65	(0.57–0.73)	46.5	0.55	0.71	0.60	(0.53–0.67)	43.5	0.65	0.54
Total score	0.67	(0.60–0.75)	58.5	0.66	0.67	0.66	(0.59–0.72)	61.5	0.59	0.72
Walking speed	0.79	(0.72–0.86)	1.03	0.78	0.75	0.72	(0.65–0.78)	1.06	0.76	0.64
Grip strength	0.68	(0.59–0.76)	17.9	0.77	0.60	0.63	(0.56–0.70)	20.4	0.59	0.62
SMI	0.56	(0.48–0.65)	7.2	0.36	0.79	0.61	(0.53–0.68)	6.3	0.67	0.53
TUG	0.76	(0.69–0.83)	-9.29	0.82	0.60	0.67	(0.60–0.74)	-8.64	0.75	0.56

TUG is converted to negative value, and AUC is calculated

*AUC* area under the curve, *SMI* skeletal muscle index, *KCL* Kihon Checklist, *mCHS* modified version of Cardiovascular Health Study, *TUG* Timed Up and Go test, *CI* confidence interval

### Incidence of mCHS-defined or KCL-defined frailty

For the mCHS criterion, the average number of observation days was 919.6 ± 508.8 (days), the follow up rate was 47.0%, and 27 of 117 (23.1%) participants developed frailty. For the KCL criterion, the average number of observation days was 866.8 ± 501.2 (days), the follow-up rate was 48.6%, and 22 of 103 (21.4%) participants developed frailty. The incidence rates of mCHS- and KCL-defined frailty were 9.16 and 8.99 per 100 person-years, respectively.

### Survival analyses

**Fig. 2:**
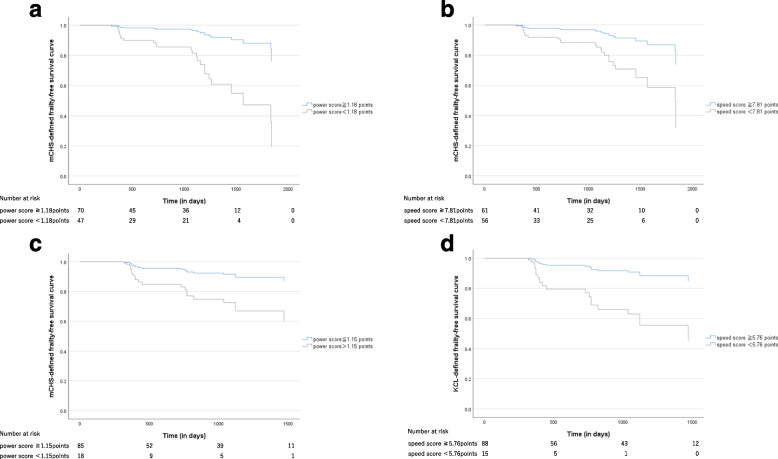
Frailty-free survival curves by groups with high and low power and speed scores according to the multivariate Cox regression analysis. The patients were classified into two groups based on the cut-off values (Yoden index) of power and speed scores. The blue and gray lines show groups with higher and lower scores, respectively. The mCHS-defined frailty-free survival curves of the two groups according to the **a** power scores or **b** speed scores and KCL-defined frailty-free survival curves of two groups according to the **c** power scores or **d** speed scores are calculated using the Cox regression analysis after adjusting for age, sex, BMI, dyslipidemia, diabetes, hypertension, heart failure, stroke, habitual drinker, and smoking. The frailty-free survivals of two groups were significantly different (**a** power score, *P* = 0.001; **b** speed score, *P* = 0.008; **c** power score, *P* = 0.014; and **d** speed score, *P* = 0.003, Cox regression analysis). BMI, body mass index

The frailty-free survival curves of the two groups with power and speed scores below and above the cutoff levels according to the multivariate Cox regression model are shown in Fig. 2. The results showed that the mCHS-defined frailty-free survival was significantly lower (a) in patients with low power scores (*P* = 0.001) and (b) in patients with low speed scores (*P* = 0.008) compared with those with high scores. The KCL-defined frailty-free survival was also significantly lower (c) in patients with low power scores (*p* = 0.014) and (d) in patients with low speed scores (*P* = 0.003).

### Multivariate Cox regression analyses

The power and speed scores significantly predicted the incidence of mCHS-defined frailty after adjusting for covariates. A 0.1-point increase in the power score and a 1-point increase in the speed score reduced the risks of mCHS-defined frailty by 55% and 28%, respectively (Table 4). Similarly, an increase in the power, speed, and total scores decreased the incidence rates of KCL-defined frailty by 44%, 23%, and 35%, respectively, although the significant association of the power score was attenuated. By contrast, the balance score was not significantly associated with mCHS-defined and KCL-defined frailty (Table 4). Patients with lower total scores had a significantly higher incidence of KCL-defined frailty (*P* = 0.028). When the MMSE scores were added as an explanatory variable in the multivariate analyses, similar results were obtained in the analysis of mCHS-defined frailty; in the analysis of KCL-defined frailty, the associations of speed and total scores with the incidence of frailty were attenuated (Supplementary Table 1).

**Table 4:** Cox regression analysis of the incidence of frailty defined based on the mCHS and KCL scores of physical performance during sit-to stand motion

	Crude model		Multivariate model	
	Odds ratio (95% CI)	*P*	Odds ratio (95% CI)	*P*
**Frailty defined by mCHS criteria**				
Power score, per 0.1-point increase	0.45 (0.25–0.78)	0.005	0.45 (0.22–0.92)	0.029
Speed score, per 1-point increase	0.69 (0.54–0.87)	0.002	0.72 (0.55–0.95)	0.020
Balance score, per 1-point increase	0.99 (0.96–1.02)	0.446	1.01 (0.97–1.05)	0.644
Total score, per 10-points increase	0.66 (0.49–0.89)	0.006	0.76 (0.52–1.11)	0.151
**Frailty defined by KCL criteria**				
Power score, per 0.1-point increase	0.77 (0.46–1.32)	0.346	0.56 (0.27–1.14)	0.110
Speed score, per 1-point increase	0.79 (0.62–0.99)	0.045	0.77 (0.60–0.99)	0.039
Balance score, per 1-point increase	0.98 (0.95–1.01)	0.260	0.97 (0.93–1.01)	0.152
Total score, per 1- points increase	0.70 (0.51–0.96)	0.027	0.65 (0.44–0.96)	0.028

The multivariate model is adjusted for age, sex, BMI, dyslipidemia, diabetes, hypertension, heart failure, stroke, habitual drinker, and smoking

*BMI* body mass index

## Discussion

The mechanisms by which the parameters of GRF during sit-to-stand motion influence frailty among older adults need to be elucidated further. This study found that the power and speed scores were independently associated with mCHS-defined and KCL-defined frailty after adjusting for the baseline covariates.

Because standing movements can be easily performed even by disabled older adults, the evaluation using the motor function analyzer in this study is feasible to most of older adults who can stand up from the sitting position by following simple instructions. It can also evaluate the three components of GRF during sit-to-stand movement. Furthermore, this method may be superior to walking speed or TUG test as it does not require a space for walking.

Our results on the evaluation of the association between power score (F/wt) or speed score (RFD/wt) and mCHS-defined physical frailty are consistent with those of a previous study, which reported the association between RFD/wt and physical performance (TUG and 5-min walk test) [16] and between low F/wt and fall risk [15]. Furthermore, F/wt and RFD/wt can accurately detect sarcopenia [25]. The ROC analysis performed in our study showed that the power score predicted the presence of KCL-defined frailty more accurately than the speed and balance scores. The power score reflects muscle strength during sit-to-stand motion, which is an important component of CGA-based frailty and physical frailty.

In our study, the balance scores were not associated with the development of frailty. This result is not consistent with that of other studies, which showed an association between balance and frailty [26, 27]. This discrepancy may be attributed to the differences in the methods used for assessing balance ability or study populations. Many methods used for assessing balance, such as time spent standing on one leg or swaying while walking, might not be accurate as they are affected by muscle strength.

In the current study, the AUC value of the power and speed scores for discriminating mCHS-defined frailty was > 0.7. In previous studies, the AUC values of standing movements were 0.76–0.81; walking speed, 0.81–0.92; and TUG, 0.87; this finding suggests that the AUC values of standing movements tend to be lower than those of walking speed and TUG time [10–12]. As this study used a device that quantifies standing movements, the predictive value of the power and speed scores for mCHS-defined frailty may have been high, as observed in the TUG test. The AUC value of power scores for KCL-defined frailty was 0.68, which was slightly lower than that of walking speed and similar to those of other indices. In the current study, the longitudinal analysis showed that only the power and speed scores predicted the incidence of frailty in patients with mCHS-defined frailty, which was consistent with the results of the cross-sectional analysis at baseline. The association observed between speed score, a measure of RFW/wt, and KCL-defined frailty suggests that instantaneous force other than the maximum GRF during sit-to-stand motion is an important risk factor for frailty. The speed score measures the length of time it takes for the GRF to reach its maximum when rising from a chair, which can also be described as an instantaneous force. The fast-twitch muscles fibers, which exert instantaneous force, diminish with age [28]. These results suggest that resistance exercises that improve muscle strength during sit-to-stand motion are important for preventing frailty. Some factors could explain the discrepancy in the results between the incidence of mCHS-defined and KCL-defined frailty (Table 4). mCHS-defined frailty is a type of physical frailty whose diagnostic criteria include muscle strength. Thus, power should be strongly related to this condition. The significant association between total score and KCL-defined frailty could be attributed to the relative stronger contribution of balance on its diagnosis compared with mCHS-defined frailty, since the KCL questionnaire include some questions related to fall and fear of fall.

When the MMSE scores were added as an explanatory variable in the multivariate Cox regression analyses, similar results were obtained for mCHS-defined frailty; in the analysis of KCL-defined frailty, the associations of speed and total scores with the incidence of frailty were attenuated. This may be because the KCL criteria include the cognitive impairment domain, with some questions evaluating the cognitive functions.

This study has some limitations. It was a single-center study. The low number of frailty events analyzed in this longitudinal study may have led to a reduction in the predictive power. The predictive ability of the sit-to-stand motion for frailty should be verified in a multicenter setting.

Second, selection bias probably exists as only patients with cardiovascular diseases were recruited for this study, which has a high risk for frailty. Third, the follow-up rate was relatively low, partly due to the ongoing coronavirus disease 2019 pandemic. However, no differences were found in almost all background data between those with and without follow-up data; the results of the analysis could represent the characteristics of the entire cohort.

Despite these limitations, our study involved both cross-sectional and longitudinal analyses, and the results revealed associations between two dynamic parameters related to the GRF and two types of frailty indices during sit-to-stand motion. The measurement of power and speed scores can provide a quantitative assessment of the GRF parameters associated with frailty within a relatively short period of time.

## Conclusion

The power and speed scores as measures of GRF during sit-to-stand motion are associated with mCHS-defined and KCL-defined frailty indices and can accurately determine frailty at baseline. Furthermore, the power score, a measure of maximum GRF divided by wt, is useful in predicting the incidence of mCHS-defined frailty. Meanwhile, the speed score, a measure of instantaneous force, is useful in predicting the incidence of two types of frailty in older adults with cardiometabolic diseases.

Since the evaluation of the indices of GRF during sit-to stand motion is convenient and does not require space, general physicians should screen patients with cardiometabolic diseases who are at high risk of frailty. Assuming that a measure of GRF is relevant to the onset of frailty and involved in its mechanism, performing exercises that increase the GRF might prevent the incidence of frailty. Moreover, further studies are necessary to examine whether the indices of GRF can be improved by exercise or whether the changes in these indices are associated with the improvement of frailty status.

## Supplementary Information

**Additional file 1.**

## Data Availability

Data of this paper is not available, but the corresponding author will consider to share with request from researchers.

## Abbreviations

AUC: Area under the curve
CGA: Comprehensive Geriatric Assessment
GRF: Ground reaction force
HDS-R: Revised Hasegawa Dementia Scale
KCL: Kihon Checklist
mCH: Modified version of the Cardiovascular Health Study
MMSE: Mini-Mental State Examination
RFD: Rate of force development
ROC: Receiver operating characteristic
SMI: Skeletal muscle index
TUG: Timed Up and Go
