# Supplementary material for: Association between physical performance during sit-to-stand motion and frailty in older adults with cardiometabolic diseases: a cross-sectional, longitudinal study
Source: BMC Geriatr. 2023 May 30;23:337. doi: 10.1186/s12877-023-04011-z (PMC10228424; doi:10.1186/s12877-023-04011-z)
Supplement: Supplementary file 1 — Additional file 1. [file 12877_2023_4011_MOESM1_ESM.docx]

**Association between physical performance during sit-to-stand motion and frailty in older adults with cardiometabolic diseases: A cross-sectional, longitudinal study**

Yuji Murao,^1^ Joji Ishikawa,^1,2^ Yoshiaki Tamura,^1,3^ Fumino Kobayashi,^1^ Ai Iizuka,^1,5^ Ayumi Toba,^1,2^ Kazumasa Harada,^1,2^ and Atsushi Araki^1,3^

^1^Center for Comprehensive Care and Research for Prefrailty, Tokyo Metropolitan Geriatric Hospital, Tokyo, Japan

^2^Department of Cardiology, Tokyo Metropolitan Geriatric Hospital, Tokyo, Japan

^3^Department of Diabetes, Metabolism, and Endocrinology, Tokyo Metropolitan Geriatric Hospital, Tokyo, Japan

**Corresponding author:**

Joji Ishikawa

35-2 Sakaecho, Itabashi-ku, Tokyo 173-0015, Japan

Tel: +81-3-3964-1141

Fax: +81-3-3964-1392

E-mail: [joji_ishikawa@tmghig.jp](mailto:joji_ishikawa@tmghig.jp)

**Supplementary Figure 1a. AUC curves of each variable for the discrimination of mCHS-defined frailty**

ROC curves for power score (solid line), speed score (dotted line), walking speed (dot-dashed line), TUG time (dashed line), and SMI (dot-dot-dashed line) for the discrimination of mCHS-defined frailty. The area under the ROC curve showed a total score of 0.67, speed score of 0.73, walking speed of 0.79, SMI of 0.56, and TUG time of 0.76.

ROC, receiver operating characteristics; mCHS, modified version of Cardiovascular Health Study; SMI: skeletal muscle index; TUG: Timed Up and Go; AUC, area under the curve


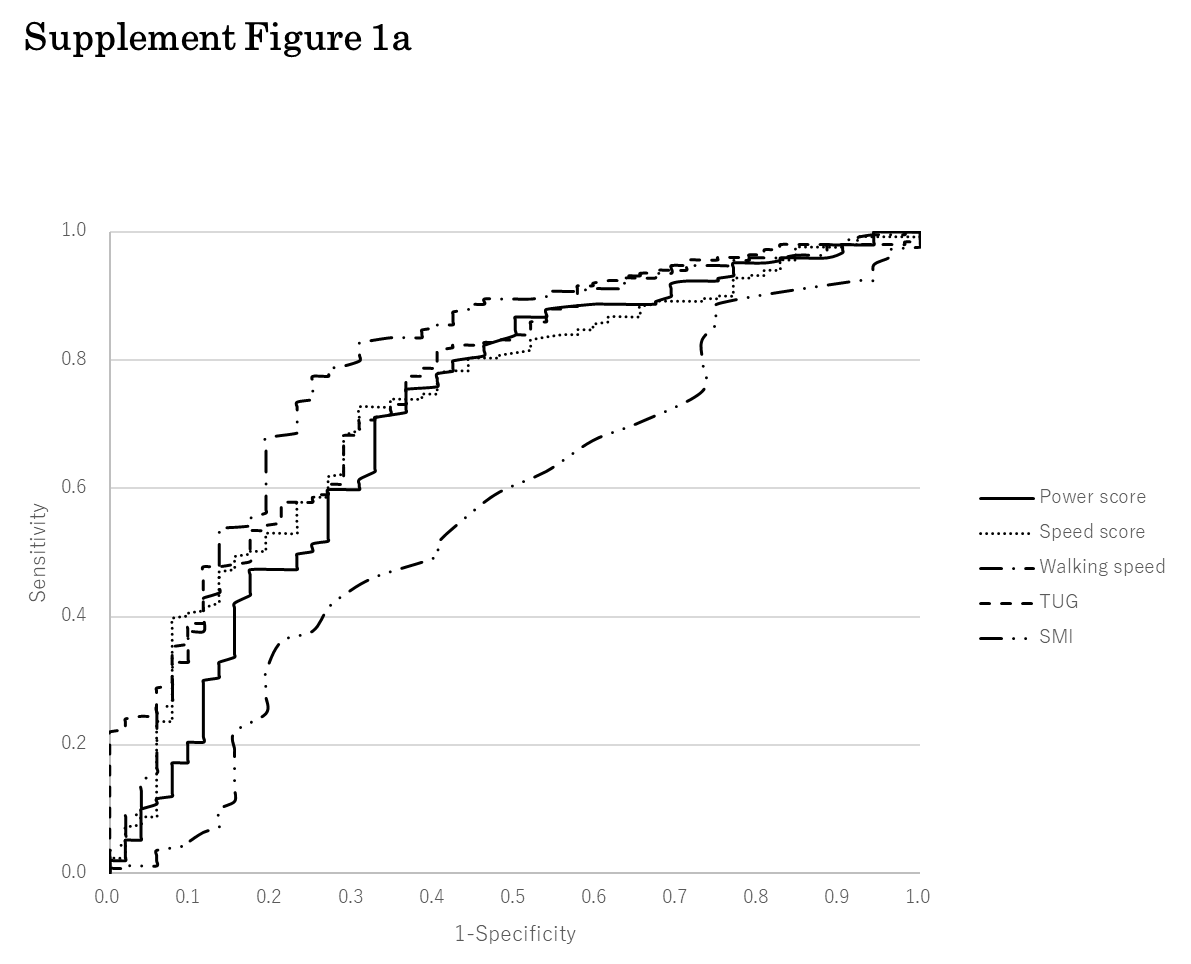


**Supplementary Figure 1b. AUC curves of each variable for the discrimination of KCL-defined frailty**

ROC curves of the power score (solid line), speed score (dotted line), walking speed (dot-dashed line), TUG time (dashed line), and SMI (dot-dot-dashed line) for the discrimination of KCL-defined frailty. The area under the ROC curve showed a total score of 0.66, power score of 0.68, walking speed of 0.72, SMI of 0.61, and TUG time of 0.67.

ROC, receiver operating characteristics; SMI: skeletal muscle index; TUG: Timed Up and Go; AUC, area under the curve; KCL, Kihon checklist


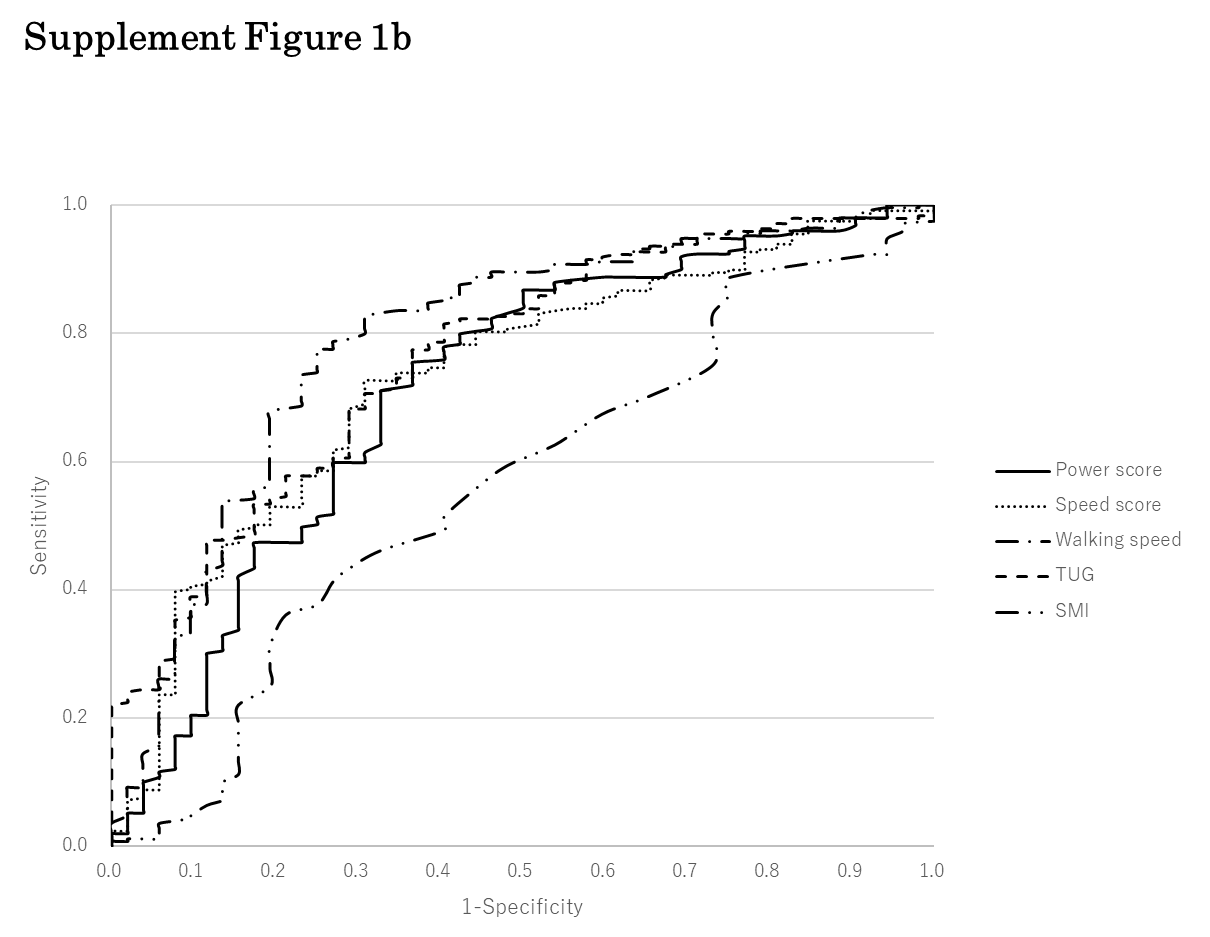


| **Supplementary Table: Cox regression analysis of the incidence of frailty defined based on the mCHS and KCL scores of physical perfomance during sit-to stand movement** | | | | | |
| --- | --- | --- | --- | --- | --- |
|  | Crude model | |  | Multivariate model | |
|  | Hazard ratio (95% CI) | P |  | Hazard ratio (95% CI) | P |
| **Frailty defined based on the mCHS criteria** |  |  |  |  |  |
| Power below vs. above 1.16 points | 1.96 (0.90–4.30) | 0.091 |  | 1.33 (0.50–3.55) | 0.567 |
| Speed below vs. above 6.92 points | 3.80 (1.73–8.35) | 0.001 |  | 3.87 (1.39–10.75) | 0.009 |
| Balance below vs. above 46.5 points | 1.43 (0.65–3.13) | 0.369 |  | 0.75 (0.28–2.02) | 0.574 |
| Total score below vs. above 58.5 points | 1.78 (0.81–3.88) | 0.149 |  | 0.77 (0.26–2.27) | 0.633 |
|  |  |  |  |  |  |
| **Frailty defined based on the KCL criteria** |  |  |  |  |  |
| Power below vs. above 1.16 points | 1.92 (0.84–4.40) | 0.121 |  | 2.38 (0.90–6.31) | 0.081 |
| Speed below vs. above 7.13 points | 2.16 (0.96–4.84) | 0.062 |  | 2.42 (0.96–6.10) | 0.060 |
| Balance below vs. above 43.5 points | 1.97 (0.87–4.42) | 0.102 |  | 2.53 (0.99–6.44) | 0.053 |
| Total score below vs. above 61.5 points | 2.40 (1.06–5.44) | 0.036 |  | 2.80 (1.00–7.88) | 0.051 |
| The multivariate model is adjusted for age, sex, BMI, dyaslipldemia,diabetes,hypertension,heart failure,stroke,habitual drinker, and smoking. | | | | | |
| BMI, body mass index; CI, confidence interval |  |  |  |  |  |
